# Visual impairment in children with cerebral palsy: Croatian population-based study for birth years 2003-2008

**DOI:** 10.3325/cmj.2019.60.414

**Published:** 2019-10

**Authors:** Neda Striber, Katarina Vulin, Ivana Đaković, Iva Prvčić, Vlasta Đuranović, Branimir Cerovski, Sanja Pejić Roško, Dunja Čokolić Petrović, Sunčica Martinec, Barbara Dawidowsky, Vlatka Mejaški Bošnjak

**Affiliations:** 1 Children's Hospital Zagreb, Zagreb, Croatia; 2 University Hospital Center Zagreb, Zagreb, Croatia; 3 University Hospital Center Osijek, Osijek, Croatia; 4 Special Hospital for Medical Rehabilitation Krapinske Toplice, Krapinske Toplice, Croatia

## Abstract

**Aim:**

To evaluate visual impairment (VI) in children with cerebral palsy (CP).

**Methods:**

This population-based study included 419 children from the Surveillance of Cerebral Palsy in Europe (SCPE) C28 RCP-HR – Register of Cerebral Palsy of Croatia born 2003-2008. Vision in children with CP (according to SCPE) was classified as normal or impaired, with the subcategory of severe VI. The proportion of children with VI was assessed in groups with different CP type/subtype, gross and fine motor function, and gestational age (GA).

**Results:**

A total of 266 children had some degree of VI (266/400; 66.5%), 134 had normal vision, and data on VI were unknown for 19 children. Severe VI was present in 44 children (44/400; 11%). The proportion of children with VI and severe VI increased with the Gross Motor Function Classification System and Bimanual Fine Motor Function levels. Children with bilateral spastic CP had the highest frequency of severe VI (14.9%). The percentage of severe VI in children with bilateral spastic CP was 53.8% in the group born <28 weeks of GA, 13.3% in the group born 28-31 weeks of GA, 11.1% in the group born 32-36 weeks of GA, and 24.4% in the group born >36 weeks of GA (λ^2^ = 4.95; df = 6; *P* < 0.001).

**Conclusion:**

Children with CP have a high prevalence of VI and severe VI, which is increasing with the level of motor impairment. Severe VI is significantly more common in children with bilateral spastic CP, especially among extremely premature infants.

Cerebral palsy (CP) is a group of non-progressive, but not unchanging, disorders of movement and/or posture and motor function, caused by a non-progressive lesion or abnormality of the developing brain. Recently, Surveillance of Cerebral Palsy in Europe (SCPE) classified CP based on neurological symptoms to spastic (bilateral and unilateral), dyskinetic (dystonic and choreo-athetotic), and ataxic (1,2).

The timing, type, and extent of the lesions are assessed by neuroimaging. Brain maldevelopments mostly occur in the first and second trimester, white matter lesions between the 24th and 34th week of gestation, and lesions of the basal ganglia and cortex after that period (3,4). In addition, the common cause of neurodevelopmental disorders in children, including CP and visual disorder, is hypoxic-ischemic lesion of periventricular white matter in preterm, ie, periventricular leukomalacia (PVL) (5-7). PVL represents a white matter injury (typical for preterms) located near the lateral ventricles, mostly in the frontal and peritrigonal areas. The latter affects the posterior limb of the capsula interna, including optical radiation, and leads to a more severe motor disability, with associated impairments, particularly visual impairment (VI) (5,7-9).

Despite the fact that CP associated with VI was first mentioned already in 1834 by Little, reports on VI in children with CP are scarce. Previous studies have shown that the frequency of VI in children with CP ranges from 35 to 85% (10-15). According to Dutton and Jacobson (16), up to 40% of the brain is involved in the complex processes of visual functioning, explaining the high prevalence and diversity of VI in children with CP. Children with CP most frequently have strabismus, but also refraction anomalies (hypermetropia, myopia, and astigmatism), reduced visual acuity, amblyopia, accommodation impairments, retinopathies, nystagmus, and cerebral VI (7,10-13,17). Different types of CP are associated with different visual disorders: spastic CP with oculomotor impairments, strabismus, and refraction anomalies; athetotic CP with refraction anomalies; and ataxic CP with nystagmus (10-13,17,18).

The aim of this population-based study was to evaluate the frequency of VI in children with CP and establish the level of functional deficit measured by Gross Motor Function Classification System (GMFCS) and Bimanual Fine Motor Function (BFMF) with regards to VI and severe VI. Furthermore, the association of gestational age (GA) and VI severity was investigated.

## PATIENTS AND METHODS

This study used the data from SCPE National Register C28 RCP-HR – Register of Cerebral Palsy in Croatia (born 01/01/2003 to 31/12/2008) collected in the period 2012-2017. The register data are collected on the county level, and the area of coverage depends on the birth year, with increasing number of counties included each year. According to the Croatian Bureau of Statistics, during 2003-2008 period in the counties included in the register, Croatian residents gave birth to 194 314 children. A total of 445 of these children were diagnosed with CP, giving the prevalence of CP of 2.29/1000. Data were collected by the county coordinator – referring neuropediatrician and/or physiatrist employed in general hospitals and/or neurorehabilitation centers in the Croatian health care system in the area covered by the register. The children with postneonatal cause of CP, children who died between the age of 24 months and the registration date, and children with the residence outside the area at the time of registration (26 children) were excluded from the study.

### Methods

Clinical data for the Register were collected from the medical records or clinical assessment records when the children were around 7 years old. Data on the CP type and subtype, GA, functional classification of GMFCS and BFMF, and accompanying VI were analyzed. CP type and subtype, as well as GMFCS and BFMF level, were assessed by a neuropediatrician and/or physiatrist.

Vision in children with CP (according to SCPE) was classified as normal or impaired (defined as any type of visual impairment). Impaired vision was classified as severe or not severe. Severe VI is defined as blindness or no useful vision after correction of a better eye, if visual acuity is <6/60 according to Snellen's table or <0.1 by decimal scale. All children with impaired vision not meeting the criteria of severe VI were considered as having not severe VI.

All activities of SCPE National Register C28 RCP-HR – Register of Cerebral Palsy in Croatia were approved by the Ethics Committee of Children’s Hospital Zagreb.

### Statistical analysis

The category distribution of patients is presented as absolute and relative frequencies. Descriptive statistics were calculated to describe the level of functionality (GMFCS, BFMF). To determine the differences in patient distribution and differences in GMFCS and BFMF between the groups we used χ^2^ test, with the level of statistical significance set to *P* < 0.05. Statistical analysis was conducted with SPSS v. 20 (IBM Corp, Armonk, NY, USA).

## RESULTS

There was no information on VI in 19/419 (4.5%) participants. A total of 266 participants had some degree of VI (266/400; 66.5%), 44 of whom had severe VI, giving the proportion of severe VI in our study of 11% (44/400). VI severity was unknown in 5.6% of children (15/266).

**Figure 1:**
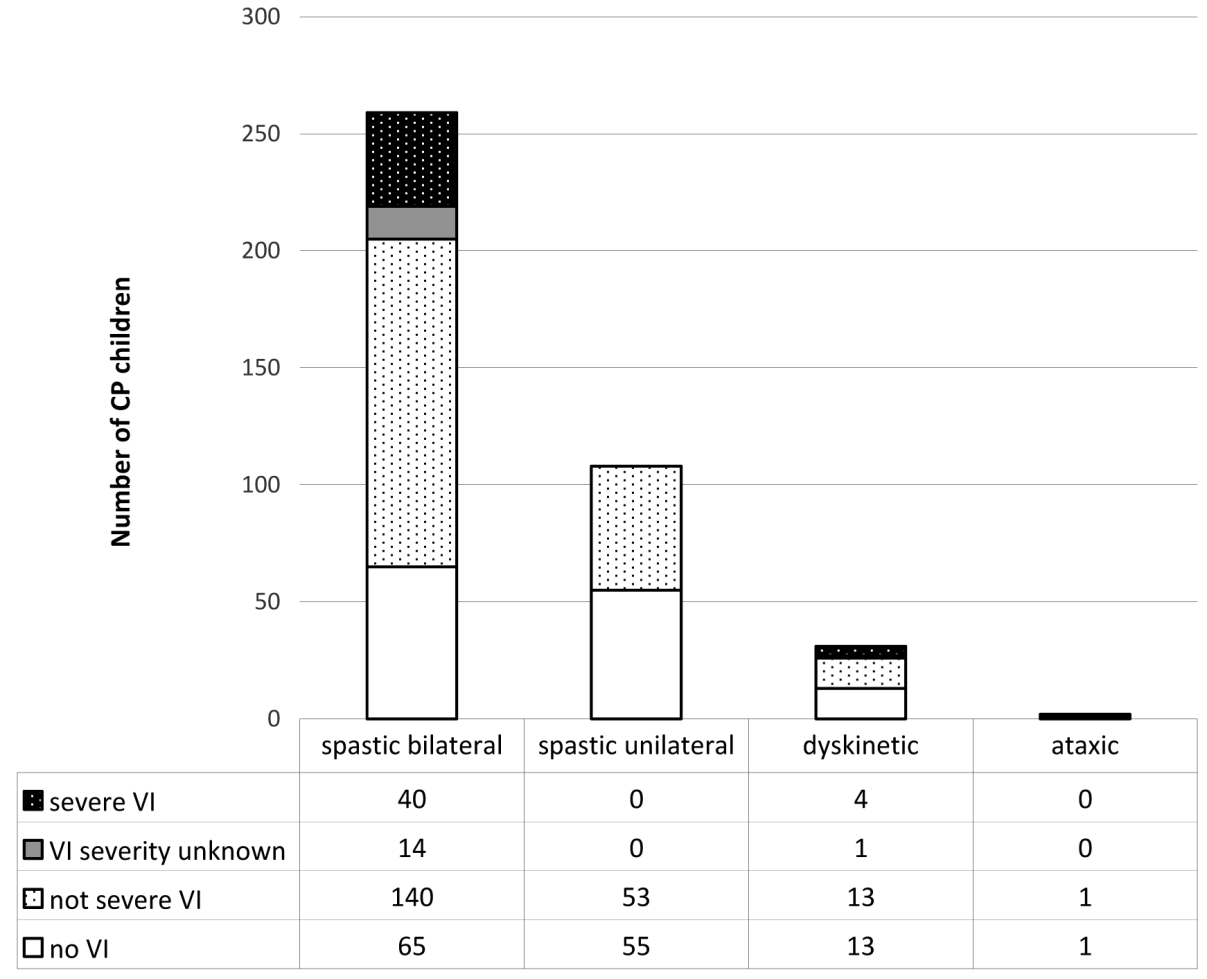
Distribution of visual impairment (VI) stratified by cerebral palsy (CP) type and subtype.

Among 266 participants with CP and VI, 247 (92.8%) had spastic (53 [19.9%] unilateral and 194 [72.9%] bilateral), 18 (6.8%) had dyskinetic, and 1 (0.4%) had ataxic CP (Figure 1).

Data were available for both VI and GMFCS levels for 398/400 participants and for both VI and BFMF levels for 388/400 participants (Table 1). Children without VI had significantly better gross motor and bimanual fine motor functionality than children with VI (GMFCS, λ^2^ = 28.21, df = 8, *P* < 0.001; BFMF, λ^2^ = 27.490, df = 8, *P* < 0.001). The same analysis was applied to patients with severe VI. In the group of 44 children with severe VI, 40 (90.9%) had spastic type (all of them had bilateral subtype) and 4 (9.1%) had dyskinetic CP. Children with spastic CP had significantly higher proportion of severe VI (λ^2^ = 19.42; df = 6; *P* < 0.005).

**Table 1:** Descriptive parameters for GMFCS and BFMF*

Functional classification	VI presence and severity	Number of CP-children	Median	Mode
GMFCS	no VI	133	2	2
	VI present	265	3	5
	not severe VI	206	3	1
	severe VI	44	5	5
BFMF	no VI	131	2	1
	VI present	257	2	2
	not severe VI	201	2	2
	severe VI	43	5	5

*CP – cerebral palsy, GMFCS – Gross Motor Function Classification System, BFMF – Bimanual Fine Motor Function, VI – visual impairment.

Children without severe VI had significantly better gross motor and bimanual fine motor functionality than children with severe VI (GMFCS, λ^2^ = 33.72, df = 8, *P* < 0.001; BFMF, λ^2^ = 29.23, df = 8, *P* < 0.001).

**Figure 2:**
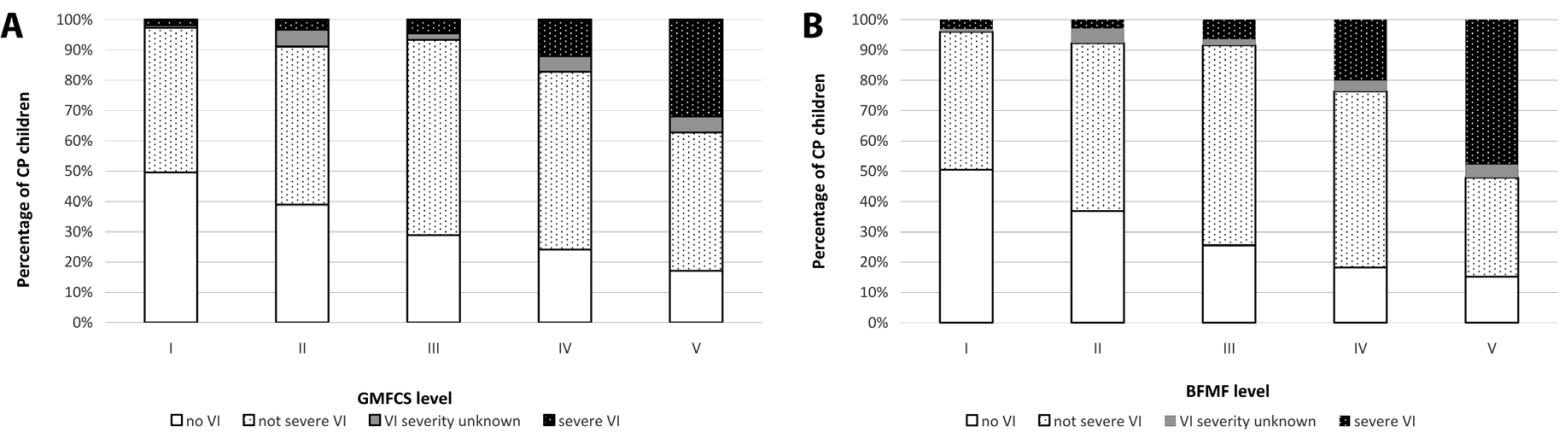
(**A**) Distribution of visual impairment (VI) stratified by Gross Motor Function Classification System (GMFCS) level. (**B**) Distribution of VI stratified by Bimanual Fine Motor Function (BFMF) level.

**Figure 3:**
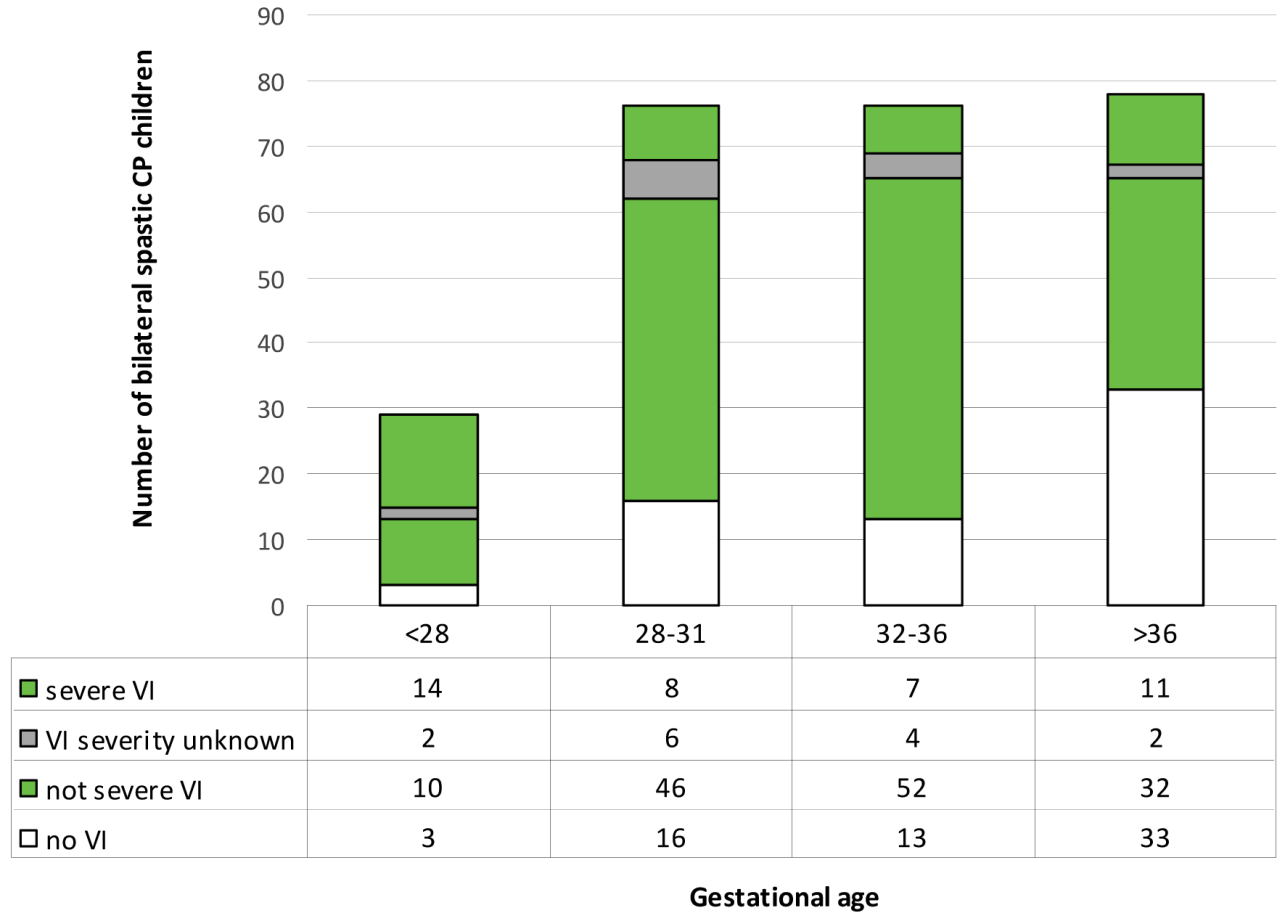
Distribution of visual impairment (VI) for children with spastic bilateral cerebral palsy (CP) stratified by gestational age.

The proportion of children with VI and severe VI gradually increased in the GMFCS and BFMF levels I-V (Table 2) (Figure 2 A,B). Children with severe VI had significantly higher GMFCS and BFMF scores (λ^2^ = 45.84, df = 8, *P* < 0.001; λ^2^ = 64.41, df = 8, *P* < 0.001, respectively). Children with bilateral spastic CP had the highest frequency of severe VI (14.9%) (Figure 1). They were divided in four groups according to GA: children born <28 weeks, 28-31 weeks, 32-36 weeks, and >36 weeks of gestation. The relative percentage of severe VI was 53.8% in children born <28 weeks of GA, 13.3% in in children born 28-31 weeks of GA, 11.1% in children born 32-36 weeks of GA, and 24.4% in in children born >36 weeks of GA (Figure 3). Children with the lowest GA had significantly more VI (λ^2^ = 4.95; df = 6; *P* < 0.001).

**Table 2:** Visual findings stratified by GMFCS and BFMF levels*

	No (%) of patients			
	with VI			without VI
	not severe VI	severe VI	severity unknown	
GMFCS				
I	53 (13.32)	2 (0.5)	1 (0.25)	55 (13.82)
II	47 (11.81)	3 (0.75)	5 (1.25)	35 (8.79)
III	29 (7.29)	2 (0.5)	1 (0.25)	13 (3.27)
IV	34 (8.54)	7 (1.76)	3 (0.75)	14 (3.52)
V	43 (10.8)	30 (7.54)	5 (1.25)	16 (4.02)
BFMF				
I	45 (11.6)	3 (0.77)	1 (0.26)	50 (12.89)
II	78 (20.1)	4 (1.03)	7 (1.8)	52 (13.4)
III	31 (7.99)	3 (0.77)	1 (0.26)	12 (3.09)
IV	32 (8.25)	11 (2.84)	2 (0.52)	10 (2.58)
V	15 (3.87)	22 (5.67)	2 (0.52)	7 (1.8)

*GMFCS – Gross Motor Function Classification System, BFMF – Bimanual Fine Motor Function, VI – visual impairment.

### DISCUSSION

Our results showed that children with CP had a high prevalence of VI and severe VI, which increased with the level of motor impairment. This study estimated the prevalence of CP to be 2.29/1000, which is in agreement with previously published population studies (14,19-21). The distribution of CP types and subtypes, as well as motor impairment severity, were also similar to those in previous reports (14,15,19). Consequently, our sample is fairly representative for the CP population.

VI is a well-known accompanying impairment in children with CP, which differs according to CP type (6,9,18,22,23). There are limited data about its prevalence in the population with CP, especially regarding to the severity of VI, due to the heterogeneity of the studies and different definitions of severe VI.

In our study, the VI proportion (66.5%) was higher than in the reports from Australian CP Register and United Kingdom CP Registers (36% and 40%, respectively) (14,15). All studies used a similar definition of VI, which encompassed any type or degree of VI. Smaller studies reported higher VI prevalence in patients with CP (9,18).

Several studies so far have investigated the correlation between neurological impairment and VI in population with CP. One study reported no association between the severity of motor impairment and the degree of refractive error in CP (22), while another cross-sectional study showed that children with severe CP were at the greatest risk for high myopia, absence of binocular fusion, dyskinetic strabismus, and severe gaze disorder (23).

We found that children with more severe functional neuromotor grading were more likely to have accompanying VI. The same was shown in Australian CP register (15).

While poor motor function might be a consequence of VI, it could also hamper the normal development of the ophthalmic system (24,25). Because patients with CP depend more than unaffected controls on visual information for balance, posture, and muscle tone, proper visual correction could additionally foster their neurorehabilitation (26-28).

Previous studies estimated that 5.5%-11% of children with CP had severe VI (14,15), while the prevalence in our study was 11%. Although it is possible that some patients were erroneously assumed to have severe VI because of motor and intellectual deficit, Novak et al (29) in their large meta-analysis reported that 1 in 10 children suffering from CP were blind. In our study, children with bilateral spastic CP had the highest frequency of severe VI, which is in accordance with bilateral occipital PVL as the most common cause of bilateral spastic CP.

There is a well-known relationship between prematurity and CP, however, only a recently performed Moldavian study further classified VI according to GA in children with CP (30). In our study, extremely preterm infants with bilateral spastic CP showed the highest prevalence of severe VI (53.8%). However, due to the small sample size, we were unable to statistically analyze the prevalence of severe VI according to GA for other CP subtypes. The Moldavian study reported somewhat lower prevalence of severe VI in extremely preterm infants (40%), however it was still the highest among all GA groups. One should also take into account the higher mortality rate in the same group due to limited access to advanced neonatal care.

The strength of this study is in a large population-based design. According to our knowledge, this is the first study of the prevalence of CP with relation to VI among Croatian children. However, our study also has several limitations, common for register-based studies. Our register contains no detailed data on the type of VI, since it collects data on diverse aspects of CP and accompanying impairments. Second, data were collected from general hospitals and neurorehabilitation centers in the Croatian health care system in the area covered by the register. Therefore, it is possible that children with minor functional deficit were omitted, possibly underestimating the CP prevalence.

Data from our register showed that children with CP had a high prevalence of accompanying VI. Since vision is one of the cornerstones of neurodevelopment, it is important to recognize and define VI in order to accomplish the best results by habilitation by visual correction and improve the overall quality of life, especially in preterm children with bilateral spastic CP. Another important point is to emphasize a multidisciplinary approach to children with CP and the role of ophthalmologist in this approach.
